# The efficacy of transcutaneous electrical acupoint stimulation on postoperative nausea and vomiting after laparoscopic surgery: a meta-analysis of randomized controlled trials

**DOI:** 10.3389/fmed.2026.1730188

**Published:** 2026-03-03

**Authors:** Sifan Qin, Jiang Liu, Jinfang He, Yun Liu, Jing Liu, Liwei Wang, Shirong Fang

**Affiliations:** 1 Shandong Second Medical University, Shandong, China; 2 Weifang People's Hospital, Shandong, China

**Keywords:** laparoscopic surgery, meta-analysis, PONV, postoperative nausea and vomiting, teas, transcutaneous electrical acupoint stimulation

## Abstract

**Background:**

Postoperative nausea and vomiting (PONV) is a common complication after laparoscopic surgery, which may cause fluid and electrolyte imbalance and delay postoperative recovery. Pharmacological interventions are only partially effective and have adverse effects. This study aimed to systematically evaluate the efficacy of transcutaneous electrical acupoint stimulation (TEAS) in managing PONV after laparoscopic surgery.

**Methods:**

We systematically review Cochrane Library, PubMed, Embase and Web of Science for randomized controlled trials (RCTs). Two investigators independently conducted study selection, data extraction, and bias assessment using the Cochrane Risk of Bias Tool. Meta-analyses were performed in RevMan 5.4 and heterogeneity was assessed using *I*^2^. Outcomes included PONV, postoperative nausea (PON), postoperative vomiting (POV), time to first flatus, and antiemetic rescue requirements.

**Results:**

Nine RCTs involving 2,550 participants (1,272 in the TEAS group and 1,278 in the control group) were analyzed. TEAS significantly reduced PONV incidence [*RR* = 0.78, 95% CI (0.70, 0.87); *P* < 0.001], PON incidence [*RR* = 0.69, 95% CI (0.67, 0.82); *P* < 0.001] and POV incidence [*RR* = 0.57, 95% CI (0.43–0.78); *P* < 0.001]. The intervention shortened the time to first flatus after surgery [MD = −3.36 h, 95% CI (−6.65, −0.21); *P* = 0.04] and decreased the use for antiemetic rescue medication [*RR* = 0.67, 95% CI (0.52, 0.87); *P* = 0.002].

**Conclusions:**

Our findings suggest that TEAS may be an effective adjunctive non-pharmacological treatment for PONV in patients undergoing laparoscopic surgery. When used in combination with standard antiemetic prophylaxis, TEAS can further reduce the incidence of nausea and vomiting, decrease the need for rescue antiemetics, and shorten the time to first flatus, thereby demonstrating significant added value in facilitating postoperative recovery.

**Systematic Review Registration:**

identifier [CRD42024560238].

## Introduction

PONV is one of the common complications after surgery, and the incidence of PONV is approximately 20%−30% after general surgery and up to 60%−80% in high-risk individuals (1, 2). PONV can lead to electrolyte imbalance, aspiration pneumonia and increased intracranial pressure. It may also prolong the length of hospital stay and increase the economic burden (3, 4). Despite the application of antiemetic medications and various novel therapies in recent years, these medications are only partially effective (5–7). Thus, PONV remains an important problem in the perioperative period.

Laparoscopic surgery has gained widespread clinical adoption due to its minimally invasive nature and rapid postoperative recovery. However, the procedure often necessitates pneumoperitoneum, which increases intra-abdominal pressure and may induce gastroesophageal reflux. Elevated intra-abdominal pressure may also stimulate the vagus nerve, thereby activating the vomiting center (8). Many antiemetic medications have been used to prevent PONV after surgery including 5-hydroxytryptamine-3 (5-HT3) receptor antagonists, neurokinin-1 (NK-1) inhibitors and dopamine receptor antagonists (9). In Western medicine, the primary mechanism is that opioids inhibit gastrointestinal motility through excitation of μ receptors in the central and gastrointestinal tract, resulting in decreased intestinal motility (10). However, monotherapy has certain limitations, and some patients with simple application of antiemetic medications still have unbearable PONV. Studies have shown that the failure rate of treatment with conventional antiemetic medications among obese patients can be as high as 45% (11, 12). The incidence rate of PONV is still around 20% in high-risk patients treated with a combination of dexamethasone and ondansetro (13). Due to the limitations of drug therapy for PONV, it is imperative to explore more non-pharmacological approaches to prevent PONV.

TEAS is a non-invasive treatment method derived from traditional Chinese acupuncture, in which electrodes are placed on acupoints to deliver electrical stimulation that induces the *de qi* sensation. It combines effects of peripheral nerve stimulation with the therapeutic principles of acupuncture (14). TEAS, based on traditional Chinese medicine acupuncture theory, has the advantages of non-invasion, convenient operation, few side effects and significant therapeutic effects. The mechanism by which TEAS can alleviate PONV may be by regulating autonomic homeostasis, correcting autonomic dysfunction, inhibiting catecholamine release and reducing gastrointestinal oedema (15). In addition, we selected the intervention was TEAS and excluded electro acupuncture (EA) because EA uses acupuncture, whereas TEAS uses electrode patches, which may affect the accuracy and robustness of the results.

There have been meta-analyses confirming the effectiveness of TEAS on nausea and vomiting after abdominal surgery under general anesthesia (16, 17). However, studies focused specifically on laparoscopic surgery have yielded conflicting results (16, 18), and there remains a lack of strong, up-to-date clinical evidence. Furthermore, previous reviews have often overlooked a critical analysis of the TEAS intervention parameters (e.g., time, frequency, intensity) and have not consistently evaluated TEAS as an adjunctive therapy to modern antiemetic protocols. Therefore, we conducted this updated systematic review and meta-analysis to assess the adjunctive effectiveness of TEAS in preventing PONV after laparoscopic surgery and to summarize the characteristics of the intervention protocols used. Therefore, we systematically searched for published articles, conducted a systematic review of the evidence, and performed meta-analysis to assess the effectiveness of TEAS in preventing PONV after general anesthesia.

## Materials and methods

### Search strategy

We systematically searched the following databases from their inception to October 31, 2025: PubMed, Embase, the Cochrane Library, and Web of Science. Search each database using a combination of subject terms and free words, and link search terms using Boolean logic operators. The search was re-run prior to the final analysis to include any new relevant studies. Search each database using a combination of subject terms and free words, and link search terms using Boolean logic operators. The search terms included: (“TEAS” OR “EA” OR “TEAS”) AND (“laparoscopic surgery” OR “laparoscopy”) AND (“PONV” OR “nausea” OR “vomiting” OR “PONV”). The full search details are in Supplementary Table S1.

### Eligible criteria

We included studies based on the following PICOS framework:

- P (Population): adult patients undergoing any type of laparoscopic surgery under general anesthesia.
- I (Intervention): TEAS applied at any acupoint, regardless of stimulation parameters (frequency, intensity, duration).
- C (Comparator): sham TEAS (placebo stimulation at the same acupoints or different locations) or no TEAS intervention. In both cases, the control group could receive the same standard pharmacological antiemetic prophylaxis as the intervention group.
- (Outcomes): at least one of the following outcomes must have been reported: incidence of PONV, PON, POV, need for rescue antiemetics, or time to first flatus.
- S (Study design): RCTs.

Exclusion criteria were: (1) non-RCTs such as case reports, reviews, or observational studies; (2) studies involving EA (with needles) or other forms of acupoint stimulation; (3) animal experiments; and (4) studies from which relevant data could not be extracted.

### Quality assessment

Two researchers independently evaluated the methodological qualities using the Cochrane bias risk tool based on the following criteria: random sequence generation, allocation concealment, blinding of participants and personnel, blinding of outcome assessment, incomplete outcome and selective reporting. The risk was classified as unclear, low, or high. Any disagreements were resolved through discussion or consultation with a third author. We chose the Cochrane tool as it is the standard for assessing risk of bias in randomized trials for systematic reviews of interventions.

### Data extraction

Retrieved articles were screened by two reviewers independently. Literature that met the criteria was read in full. The two reviewers then independently extracted the following information using a pre-designed standardized data extraction form: author name, publication year, sample size, age, type of surgery, intervention measures (including TEAS parameters), control measures, and target outcomes. Data were cross-checked for accuracy, with discrepancies resolved by consensus.

### Statistical analysis

Statistical analyses were carried out using RevMan 5.4 (RevMan, the Cochrane Collaboration, Oxford, United Kingdom) software. Dichotomous and continuous variables were represented by risk ratio (RR) and mean difference (MD), respectively, with 95% confidence interval (CI). Significant heterogeneity was considered when *I*^2^ > 50% and *P* < 0.1, and a random-effects model was used for analysis (19). Otherwise, a fixed-effects model was used. To evaluate result consistency and discern heterogeneity determinants, we performed successive sensitivity tests through cyclic elimination of single studies from the analytical cohort. Heterogeneity was assessed using the Chi-squared test and the *I*^2^ statistic. Significant heterogeneity was considered when *I*^2^ > 50% and *P* < 0.1, and a random-effects model was used for analysis. Otherwise, a fixed-effects model was used. To evaluate result consistency and discern heterogeneity determinants, we performed successive sensitivity tests through the leave-one-out method, cyclically eliminating single studies from the analytical cohort. Subgroup analyses were pre-specified to explore potential sources of heterogeneity, such as the type of surgery.

## Results

### Literature search

**Figure 1:**
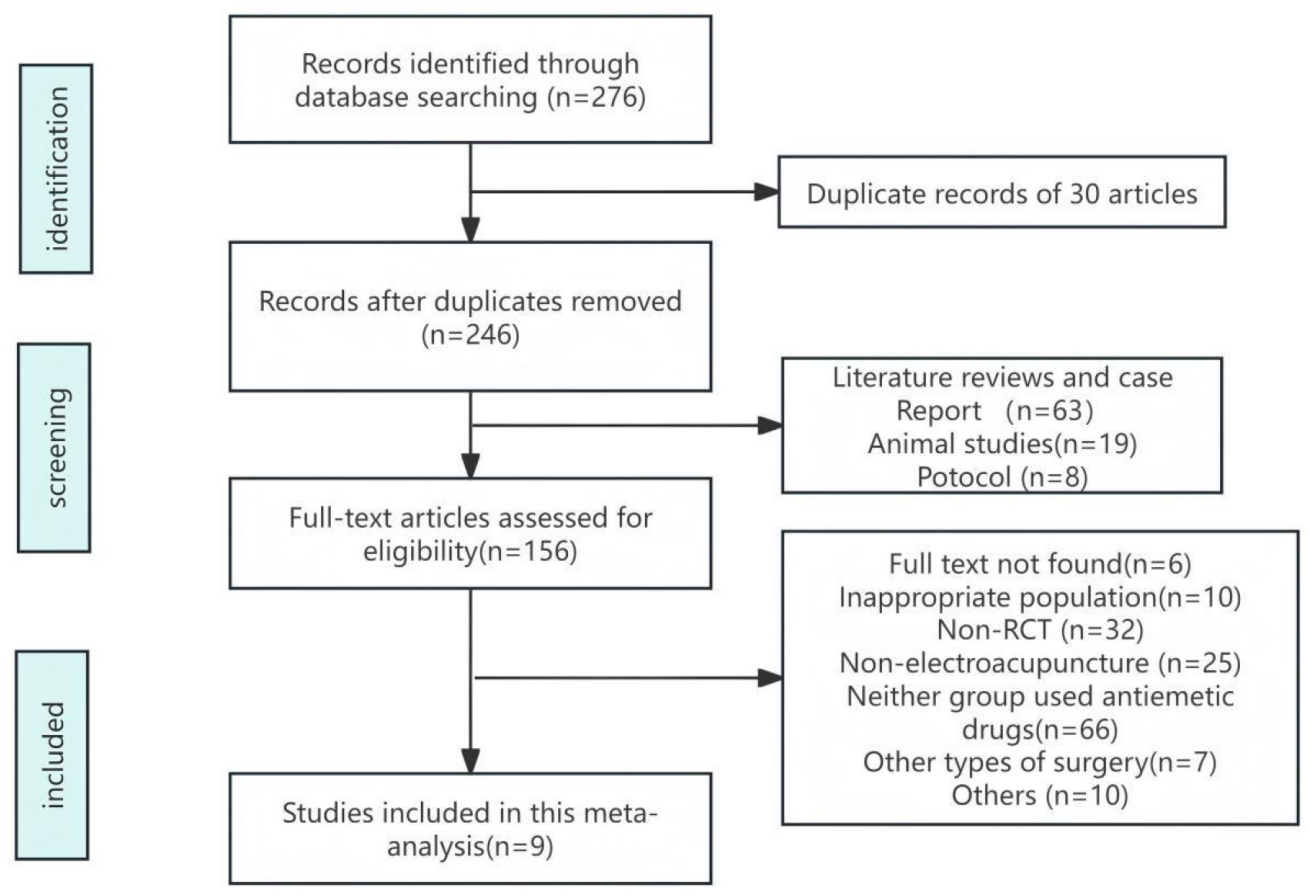
Flow diagram of the literature search.

Based on the search strategy, we identified 276 potential relevant studies in initial search. After reviewing titles and abstracts and removing duplicates, 120 records were ultimately excluded. A total of 156 full-text articles were selected for the secondary screening phase. Finally, nine studies were included in this meta-analysis (2, 20–27). The flow diagram of the study selection is shown in Figure 1.

### Study characteristics

The nine articles included in this meta-analysis were published in 2015–2025. The observation group received TEAS in nine articles, while the control group received sham TEAS in eight papers and no TEAS in one paper. In the Sham TEAS group, the patients were connected to the same acupoints, but electronic stimulation was not applied. Nine studies involved 2,550 participants, including 1,272 patients received TEAS and 1,278 patients received sham TEAS or no TEAS. The sample sizes varying from 62 to 1,655 participants. The surgical types were gynecological laparoscopic surgery, laparoscopic non-gastrointestinal surgery, laparoscopic radical gastrectomy, laparoscopic sleeve gastrectomy. Table 1 shows the characteristics of the included article.

**Table 1:** Features of the included studies.

**Reference**	**Sample size (T/C)**	**Age year (T/C)**	**Types of surgery**	**TEAS group**	**Control group**	**Acupoints**	**Target outcomes**	**Waveform**	**Frequency (Hz)**	**Current intensity (mA)**	**Single session duration (min)**	**Intervention frequency**
Gao et al. (2), 2022	1,655 (827/828)	39.0 (31.0, 46.0) 39.0 (31.0, 46.0)	Laparoscopic non-gastrointestinal surgery	TEAS + 5 mg dexamethasone + 0.075 mg palonosetron	Sham TEAS + 5 mg dexamethasone + 0.075 mg palonosetron	PC6, ST36	1, 3	Dense–disperse wave	2/100	Adjusted to patient tolerance (not specified)	30	Once before anesthesia induction
Qin et al. (25), 2023	162 (81/81)	45 ± 7.45 45 ± 5.92	GLS	TEAS + 4 mg dexamethasone + 0.25 mg palonosetron	Sham TEAS + 4 mg dexamethasone + 0.25 mg palonosetron	PC6, LI4	2, 3, 5	Dense–disperse wave	2/100	Adjusted to patient tolerance (5–12 mA)	30	Once before anesthesia induction
Xiong et al. (23), 2021	62 (31/31)	27.5 ± 8.0 27.3 ± 8.3	LSG	TEAS + 10 mg dexamethasone + 4 mg tropisetron	Sham TEAS + 10 mg dexamethasone + 4 mg tropisetron	PC6, ST36	2, 3, 4	Dense–disperse wave	2/100	Adjusted to patient tolerance (not specified)	30	Once before anesthesia induction and continued intraoperatively
Zeng et al. (24), 2023	94 (47/47)	30.5 ± 7.8 30.3 ± 6.8	LSG	TEAS + 5 mg dexamethasone + 3 mg granisetron	Sham TEAS + 5 mg dexamethasone + 3 mg granisetron	PC6, LI4	1, 4, 5	Dense–disperse wave	5/100	Adjusted to patient tolerance (not specified)	20	Once before anesthesia induction
Yan et al. (26), 2023	184 (91/93)	41.9 ± 12.4 43.6 ± 9.9	GLS	TEAS + 8 mg ondansetron	Sham TEAS + 8 mg ondansetron	LI4, PC6, ST36, SP6	1, 2, 3	Dense–disperse wave	2/100	Adjusted to patient tolerance (not specified)	30	Once before anesthesia induction
Gu et al. (22),2019	117 (58/59)	57.59 ± 7.32 56.67 ± 6.23	LRG	TEAS + 0.25 mg palonosetron	Sham TEAS + 0.25 mg palonosetron	PC6, ST36	1, 5	Dense–disperse wave	2/100	Adjusted to patient tolerance (5–30 mA)	30	Once before anesthesia induction and three times a day every day for postoperative 2 day
Yang et al. (20), 2015	100 (50/50)	37 (24–60) 35 (22–60)	GLS	TEAS + dexamethasone	Dexamethasone	PC6	1, 4, 5	Dense–disperse wave	2/100	Adjusted to patient tolerance (6- 20 mA)	30	Once before anesthesia induction and continued intraoperatively
Yao et al. (21), 2015	71 (35/36)	34.2 ± 7.2 35.6 ± 8.7	GLS	TEAS + 5mg tropisetron	Sham TEAS + 5mg tropisetron	LI4, PC6, ST36, SP6	2, 3	Dense–disperse wave	2/100	Adjusted to patient tolerance (6–9 mA)	30	Once before anesthesia induction
Pan et al. (27), 2023	105 (52/53)	42.2 ± 5.5 43.2 ± 6.3	GLS	TEAS + 5mg tropisetron	Sham TEAS + 5mg tropisetron	LI4, PC6, ST36, SP6	3, 4	Dense–disperse wave	2/100	Adjusted to patient tolerance (not specified)	30	Once before anesthesia induction and continued intraoperatively

GLS, gynecological laparoscopic surgery; LSG: Laparoscopic sleeve gastrectomy; LRG: laparoscopic radical gastrectomy.

1: postoperative nausea and vomiting; 2: postoperative nausea; 3: postoperative vomiting; 4: the incidence of requiring antiemetic rescue; 5: time to first flatus; acupoint: Neiguan PC6, Zusanli ST36, Hegu LI4, Sanyinjiao SP6.

### Risk of bias assessment

**Figure 2:**
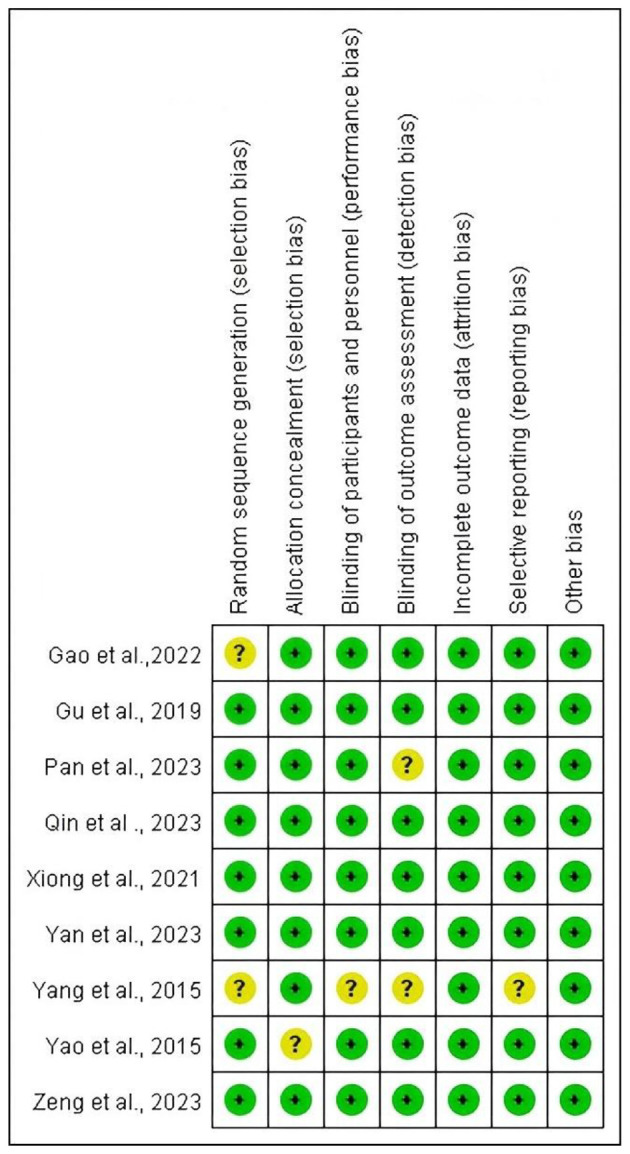
Risk of bias graph from the Cochrane risk of bias tool. Red indicates a high risk of bias, yellow indicates an unclear risk of bias, and green indicates a low risk of bias.

**Figure 3:**
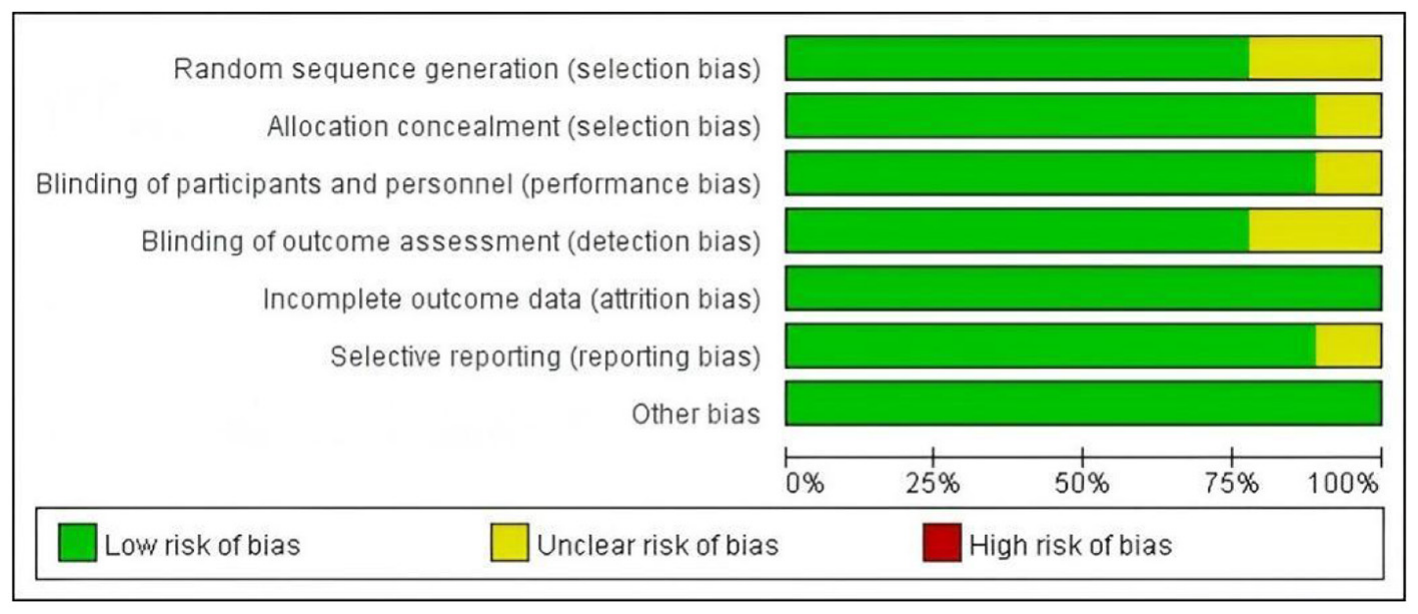
Risk of bias summary: review authors' judgments about each methodological quality item presented as percentages across all included studies. Red represents a high risk of bias, yellow represents an unclear risk of bias, and green represents a low risk of bias.

We assessed the quality of the literature using the Cochrane bias risk tool. Among the included studies, six studies (22, 23, 25–27) reported appropriate randomization and allocation concealment methods, two studies (2, 20) did not reported randomization methods and one studies (21) did not mention allocation concealment, leading to an assessment of some selection bias. One study (20) did not include a sham group. Two studies (20, 27) employed randomized designs but did not specify detailed data collection methods. No studies have incomplete outcome data. One study (20) may have selective reporting bias (Figures 2, 3).

### Incidences of PONV, PON, and POV

**Figure 4:**
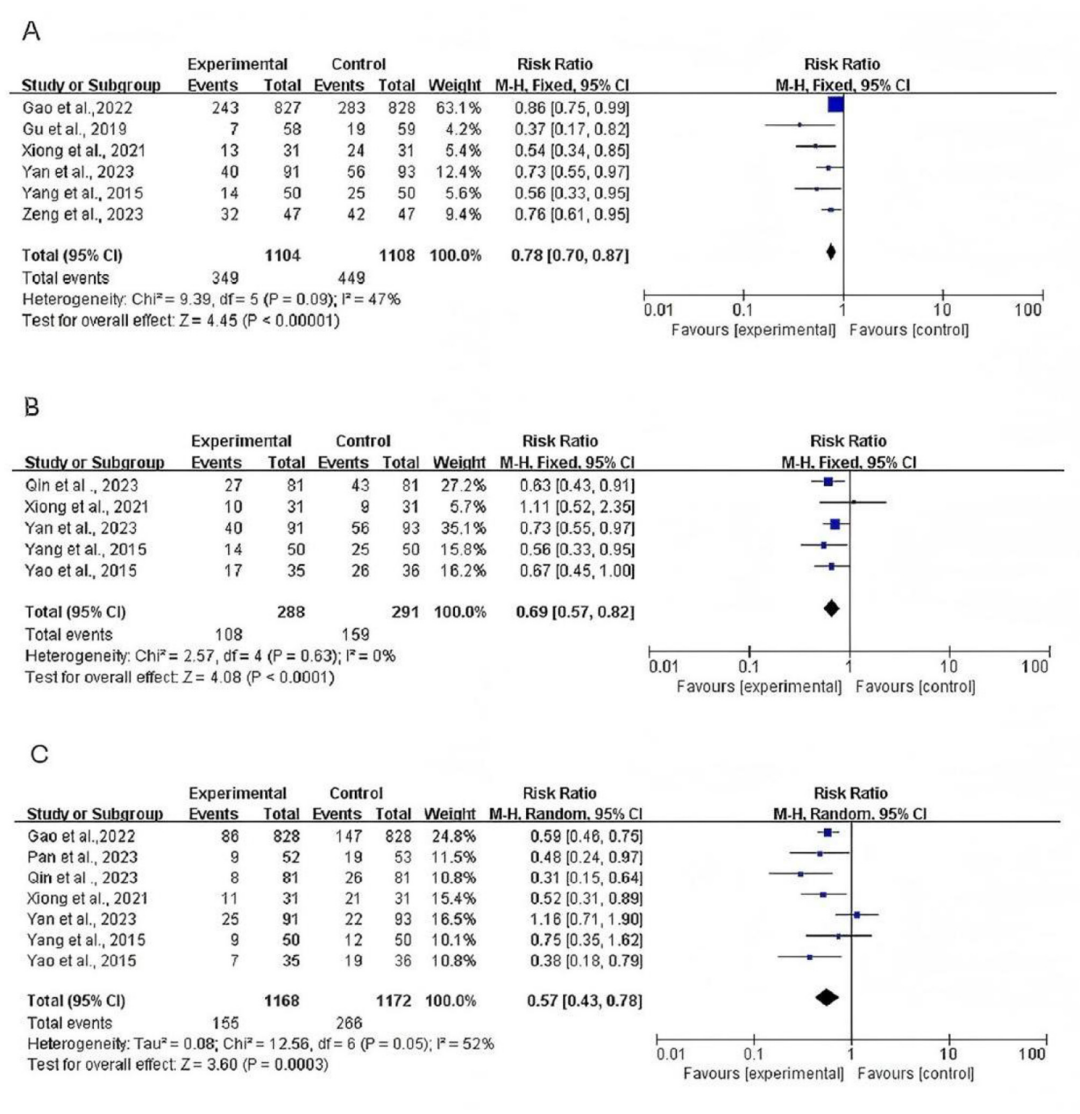
**(A)** Forest plots comparing the incidence of PONV between TEAS and Control group. **(B)** Forest plots comparing the incidence of PON between TEAS and Control group. **(C)** Forest plots comparing the incidence of POV between TEAS and Control group.

Six studies included 2,212 participants (control group 1,104 and intervention group 1,108) and measured the results of the incidence of PONV within postoperative 24 h. A fixed-effects model was used (*P* = 0.09; *I*^2^= 47%). The results indicated that participants who received TEAS exhibited a significantly lower incidence of PONV than those in the control group [*RR* = 0.78; 95% CI (0.70, 0.87); *P* **<** 0.001; Figure 4A].

Five RCTs reported the incidence of PON. There was no evidence of heterogeneity among the studies and a fixed-effects model was performed to analyze the outcome (*P* = 0.63, *I*^2^ = 0%). The meta-analysis found participants in TEAS group had lower incidence of PON [RR=0.69; 95% CI (0.57, 0.82), *P* **<** 0.001] than control group (Figure 4B).

Seven RCTs reported the incidence of POV. A random-effects model was used (*P* = 0.05, *I*^2^ = 52%). The meta-analysis also found that TEAS group has lower incidence of POV [*RR* = 0.57; 95% CI (0.43, 0.78), *P* **<** 0.001] than the control group (Figure 4C).

### Time to first flatus after surgery

**Figure 5:**
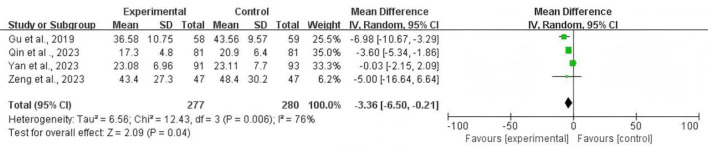
Forest plot comparing the risk ratios of the time to first flatus after surgery between the TEAS group and the control group.

Four articles including 277 patients in the TEAS group and 280 patients in the control group reported the effect of TEAS on the time of first flatus after operation. Our results showed heterogeneity among the outcome indicators (*P* = 0.006, *I*^2^ = 76%). A randomized effect model was used to analyze the outcome. Our results revealed that TEAS could significantly shorten the time to first flatus compared with the control group [MD = −3.36; 95% CI (-6.5,−0.21), *P*=0.04; Figure 5].

To explore the source of this high heterogeneity, we performed a subgroup analysis based on the type of surgery (gynecological vs. non-gynecological laparoscopic surgery). The analysis showed that the effect of TEAS was more pronounced and consistent in the non-gynecological surgery subgroup [Two studies; MD = −5.50; 95% CI (−7.15, −3.85); I^2^ = 0%] compared to the gynecological surgery subgroup [2 studies; MD = −1.20; 95% CI (-4.55, 2.15); *I*^2^ = 65%]. This suggests that the type of surgery is a significant source of heterogeneity for this outcome.

### Incidence of patients needing antiemetic rescue

**Figure 6:**
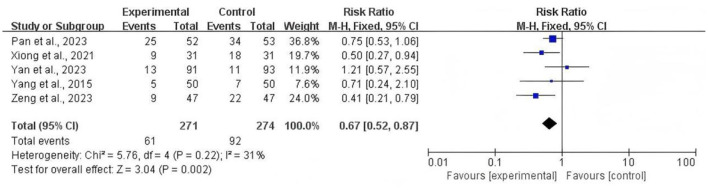
Forest plot comparing the risk ratios of the incidence of patients needing antiemetic rescue between the TEAS group and the control group.

Five studies reported the patients who required antiemetic rescue. We analyzed the results using a fixed-effects mode (*P* = 0.22; *I*^2^ = 31%). The results showed the use of rescue antiemetics was significantly lower in the TEAS group than in the control groups [RR = 0.67; 95% CI (0.52, 0.87); *P* = 0.002; Figure 6].

### Safety evaluation

None of the nine included studies reported any serious adverse events related to TEAS, such as skin infections, electrical burns, or significant pain intolerance. Some studies mentioned minor, transient skin redness at the electrode site, which resolved spontaneously. However, it is important to note that the systematic monitoring and reporting of minor adverse effects were not consistently detailed across all trials. Therefore, the absence of reported adverse events in the literature should be interpreted with caution, as it does not definitively confirm the complete absence of any potential side effects.

## Discussion

The results of data synthesis demonstrated that the application of TEAS reduced the incidences of PONV, PON, and POV, reduce the use of postoperative antiemetic drugs and shorten the time to first flatus after surgery. This suggests that TEAS confers multiple benefits during the recovery period and helps to facilitate postoperative recovery. The most commonly used acupoints across the nine trials were PC6 (nine studies), ST36 (six), LI4 (five), and SP6 (three).

Our analysis of the time to first flatus revealed high heterogeneity (*I*^2^ = 76%). To investigate this, we conducted a subgroup analysis based on the type of surgery. The results suggest that TEAS has a more consistent and significant effect in accelerating gastrointestinal recovery in patients undergoing non-gynecological laparoscopic surgery compared to those undergoing gynecological procedures. A possible explanation is that patients undergoing gynecological surgery, who are predominantly female, already have a higher baseline risk for postoperative gastrointestinal dysfunction due to hormonal influences and the specific nature of pelvic surgery. This higher baseline impairment might make the additional benefit of TEAS less pronounced or harder to detect compared to a more general surgical population. This finding highlights the importance of considering patient populations and surgical types when evaluating the efficacy of TEAS.

The precise mechanism by which TEAS alleviates PONV after laparoscopic surgery is multifactorial, but can be understood in the context of the procedure's specific physiological challenges. Laparoscopic surgery necessitates pneumoperitoneum, which increases intra-abdominal pressure and can cause significant vagal nerve stimulation, a primary trigger for PONV (8). TEAS, particularly at acupoints like PC6 and ST36, is thought to modulate autonomic nervous system activity, specifically by enhancing vagal tone in a regulatory manner (28). This may counteract the disruptive vagal stimulation from pneumoperitoneum and help restore normal gastrointestinal motility. Furthermore, the surgical stress inherent in laparoscopy triggers a neuroendocrine response, including activation of the hypothalamic-pituitary-adrenal (HPA) axis, which is linked to PONV (29). TEAS has been shown to attenuate HPA axis hyperactivity, thereby mitigating the stress response. This may be particularly relevant for the gynecological surgery patients included in our review, who are considered a high-risk population for PONV. Lastly, by stimulating Aβ and Aδ fibers, TEAS can trigger the release of endogenous opioids like endorphins in the central nervous system (30, 31), which not only contributes to analgesia but also modulates the chemoreceptor trigger zone and vomiting center. Clinical studies suggest broader perioperative benefits, including analgesia, immunomodulation, and complication reduction (31–33). In addition, the inflammatory response triggered by laparoscopic surgery may exacerbate PONV. TEAS exerts anti-inflammatory effects and may indirectly alleviate symptoms by down-regulating pro-inflammatory mediators such as IL-6 and TNF-α (34, 35).

It is also important to consider the heterogeneity within the control groups of the included studies. Eight of the nine trials employed a “sham TEAS” group, where patients were connected to a non-functional device, while one study used a “no TEAS” control. Studies using a sham TEAS control are methodologically stronger for assessing the specific physiological effects of the electrical stimulation itself, as they control for the powerful placebo effect associated with the ritual of applying electrodes and receiving attention. The inclusion of one study with a no-intervention control might slightly inflate the overall effect size. This distinction is crucial for interpreting the magnitude of the benefit attributed to TEAS and underscores the need for rigorous sham controls in future trials.

Meta-analys has demonstrated that TEAS is significantly effective in preventing PONV following general anesthesia (13). Our research indicates that TEAS effectively prevents PONV following laparoscopic surgery, consistent with previous studies. However, it is noteworthy that the study by Yan et al. (26), despite its significant weight in our analysis, did not show a statistically significant benefit of TEAS for the primary PONV outcome. Several factors might contribute to this discrepancy. Firstly, as the authors noted, the study population consisted exclusively of high-risk patients (Apfel score ≥ 3), where the baseline PONV incidence is exceptionally high, making it more challenging to demonstrate a significant effect from any single intervention. Secondly, the study employed a four-acupoint combination (LI4, PC6, ST36, SP6), which differs from other positive studies that used two or three acupoints. The specific synergistic or antagonistic effects of this combination warrant further investigation. Finally, the control group received standard prophylactic ondansetron, setting a high therapeutic bar for TEAS to show an additional benefit. This highlights the importance of context when interpreting results, particularly when TEAS is evaluated as an adjunctive therapy rather than a standalone one. In addition to gynecological procedures, RCTs covering other types of laparoscopic abdominal surgery were included. Thus, our study provides a more comprehensive assessment than the 2020 TEAS meta-analysis (16).

PC6 (Neiguan), located on the pericardium meridian, is effective in relieving PONV (36). According to the theory of Chinese medicine theory, the acupoints ST36 (Zusanli), located on Foot Yangming Stomach Meridian, can restore gastrointestinal function and alleviate PONV. Stimulation of ST36 can promote the recovery of gastrointestinal function and alleviate PONV (37, 38). LI4 (Hegu) is the original point of the large intestine channel of Hand Yangming Large Intestine Meridian. LI4 has been confirmed to be related to analgesic and sedative effects (39). In addition, a meta-analysis has shown that the use of LI4, PC6 and ST36 exerts a synergistic analgesic effect in laparoscopic surgery (40).

The Cochrane bias risk tool was used to assess the quality of the literature, and while most studies were rated as having a low risk of bias in key domains like randomization, we identified some articles with potential bias including selection bias, performance bias, detection bias and reporting bias, with a critical appraisal of performance bias being warranted. It is notoriously difficult to achieve effective participant and personnel blinding in TEAS trials; even when a sham device is used (i.e., identical electrode placement without electrical current), participants may discern the lack of sensation, potentially unblinding them to the intervention. This potential for unblinding constitutes a significant performance bias that could lead to an overestimation of the treatment effect due to placebo or expectation effects. Although we have reported the bias risk as assessed by the original study authors, readers should interpret the findings with caution, acknowledging that the true effect size of TEAS might be more modest than what is reported here, and this issue is a major challenge for the entire field of non-pharmacological stimulation research. The results of this meta-analysis demonstrated that the incidence of PONV and the need for remedial antiemesis within 24 h postoperatively were significantly lower in the TEAS group than in the control group. These findings align with those reported by Gao et al. (2) and Sun et al. (41), which suggest that TEAS enhances gastrointestinal function in patients undergoing general anesthesia, while notably differing from those of Zhang et al. (18), who found no significant effect of TEAS on PONV, and the discrepancy may be attributable to differences in surgical types and acupoint selection between the studies. In addition, no serious adverse events and treatment-related safety concerns related to TEAS were reported in the included studies, confirming its current safety and efficacy in clinical practice. Some patients may experience redness and itching on the skin where the surface electrode is applied, but this is not associated with any serious adverse events. Further studies are warranted to confirm the long-term safety of TEAS.

A key aspect that this meta-analysis aimed to address was the variability in TEAS intervention protocols. The most common stimulation frequency was a dense-disperse wave (2/100 Hz), utilized in the majority of trials. The intensity was typically adjusted to the patient's tolerance, usually described as a strong but non-painful sensation. The duration of stimulation also varied, with most studies applying TEAS for approximately 30 min before anesthesia induction and continuing it throughout the surgery. This lack of standardization makes it difficult to determine an optimal TEAS protocol. The heterogeneity in these parameters could contribute to the variability in treatment effects observed across studies. Future research should focus on head-to-head comparisons of different stimulation parameters to establish evidence-based guidelines for clinical practice.

In our meta-analysis, we included a larger number of patients and RCTs. Compared with previous meta-analyses, we highlight that the RCTs included herein explicitly documented the peri-operative administration of antiemetics such as dexamethasone or metoclopramide. Although the mechanism underlying the combined use of TEAS and antiemetic drugs remains unclear at present. This further supports the effectiveness of TEAS as a complementary therapy for preventing PONV.

### Limitations

This systematic review and meta-analysis has several limitations that should be considered when interpreting the results. First, despite our efforts to explore heterogeneity through subgroup analysis, significant unexplained heterogeneity remained for some outcomes, such as the time to first flatus. This suggests that other variables, not fully captured in our analysis, may be influencing the results. Second, the methodological quality of the included RCTs was variable. As discussed, the inherent difficulty of blinding in TEAS studies led to an “unclear” risk of performance bias in several trials, which could potentially inflate the observed treatment effect. Third, a significant limitation is the geographic concentration of the included evidence. All nine RCTs were conducted in China. This may limit the generalizability of our findings to other ethnic populations and healthcare systems and raises the possibility of location bias. Therefore, a degree of caution is necessary when applying these conclusions to a global patient population. Finally, the substantial variability in TEAS protocols (e.g., acupoint selection, stimulation parameters) and control group interventions (sham vs. no TEAS) across studies complicates the formulation of a specific, evidence-based clinical recommendation. While our findings support the general use of TEAS as an adjunct, the optimal method of application remains unclear. Future large-scale, multicenter, and multinational RCTs with standardized protocols are needed to address these limitations. Even in the latest guidelines on acupoint stimulation in China, there is no relevant recommendations of frequency model choice.

Compared with previous meta-analyses, this study provides several key contributions. Firstly, it offers the most up-to-date evidence synthesis by including several recent, large-scale RCTs, thereby increasing the statistical power and precision of the findings. Secondly, our study explicitly reframes TEAS as an adjunctive therapy and evaluates its “added value” on top of standard pharmacological antiemetics, which reflects current clinical practice more accurately. Finally, we provide a systematic summary of the TEAS stimulation parameters used in the included trials, offering valuable information for designing future studies and guiding clinical application, a gap identified in prior reviews.

## Conclusion

In summary, our study suggests that TEAS, when used as an adjunctive therapy to standard pharmacological prophylaxis, can significantly decrease the incidence of PONV, PON, and POV, accelerate the return of postoperative bowel function, and reduce the use of rescue antiemetics. These findings indicate that TEAS is an effective and safe complementary intervention for managing PONV in the context of laparoscopic surgery. It may be promising to promote postoperative gastrointestinal recovery. Future studies of high quality and large samples are needed to support the clinical benefits of TEAS therapy on gastrointestinal function.

## Data Availability

The original contributions presented in the study are included in the article/Supplementary material, further inquiries can be directed to the corresponding author.
