# Supplementary material for: The efficacy of transcutaneous electrical acupoint stimulation on postoperative nausea and vomiting after laparoscopic surgery: a meta-analysis of randomized controlled trials
Source: Front Med (Lausanne). 2026 Mar 3;13:1730188. doi: 10.3389/fmed.2026.1730188 (PMC12992031; doi:10.3389/fmed.2026.1730188)
Supplement: Supplementary file 1 [file Table_1.doc]

Appendix 1

| Table S1 Search strategy | |
| --- | --- |
| PubMed/Medline | |
| #1 | acupuncture[MeSH Terms] |
| #2 | moxibustion[MeSH Terms] |
| #3 | electroacupuncture[MeSH Terms] |
| #4 | acupuncture therapy[MeSH Terms] |
| #5 | acupressure[MeSH Terms] |
| #6 | transcutaneous electric nerve stimulation[MeSH Terms] |
| #7 | acupuncture[Title/Abstract] |
| #8 | moxibustion[Title/Abstract] |
| #9 | electroacupuncture[Title/Abstract] |
| #10 | acupuncture therapy[Title/Abstract] |
| #11 | acupressure[Title/Abstract] |
| #12 | transcutaneous electric nerve stimulation[Title/Abstract] |
| #13 | transcutaneous electric[Title/Abstract] |
| #14 | TEN[Title/Abstract] |
| #15 | TEA[Title/Abstract] |
| #16 | #1 OR #2 OR #3 OR #4 OR #5 OR #6 |
| #17 | #7 OR #8 OR#9 OR #10 OR #11 OR #12 OR#13 OR #14 OR #15 |
| #18 | #16 OR # 17 |
| #19 | nausea[Title/Abstract] |
| #20 | vomiting[Title/Abstract] |
| #21 | postoperative nausea[Title/Abstract] |
| #22 | postoperative vomiting[Title/Abstract] |
| #23 | retching[Title/Abstract] |
| #24 | vomit[Title/Abstract] |
| #25 | emesis[Title/Abstract] |
| #26 | PONV[Title/Abstract] |
| #27 | PON[Title/Abstract] |
| #28 | POV[Title/Abstract] |
| #29 | postoperative nausea and vomiting[Title/Abstract] |
| #30 | vomiting[MeSH Terms] |
| #31 | nausea[MeSH Terms] |
| #32 | postoperative nausea and vomiting[MeSH Terms] |
| #33 | #19 OR #20 OR #21 OR #22 OR #23 OR #24 OR #25 OR #26 OR #27 OR #28 OR #29 |
| #34 | #30 OR #31 OR #32 |
| #35 | #33 OR #34 |
| #36 | #18 AND #35 |
|  |  |
| Cochrane library | |
| #1 | MeSH descriptor: [Moxibustion] explode all trees |
| #2 | MeSH descriptor: [Acupuncture] explode all trees |
| #3 | MeSH descriptor: [Electroacupuncture] explode all trees |
| #4 | MeSH descriptor: [Acupuncture Therapy] explode all trees |
| #5 | MeSH descriptor: [Acupressure] explode all trees |
| #6 | MeSH descriptor: [Transcutaneous Electric Nerve Stimulation] explode all trees |
| #7 | MeSH descriptor: [Nausea] explode all trees |
| #8 | MeSH descriptor: [Vomiting] explode all trees |
| #9 | MeSH descriptor: [Postoperative Nausea and Vomiting] explode all trees |
| #10 | (acupuncture):ti,ab,kw OR (moxibustion):ti,ab,kw OR (electroacupuncture):ti,ab,kw OR (acupuncture therapy):ti,ab,kw OR (acupressure):ti,ab,kw |
| #11 | (transcutaneous electric nerve stimulation):ti,ab,kw OR (transcutaneous electric):ti,ab,kw OR (TEN):ti,ab,kw OR (TEA):ti,ab,kw |
| #12 | (nausea):ti,ab,kw OR (vomiting):ti,ab,kw OR (postoperative nausea and vomiting):ti,ab,kw OR (retching):ti,ab,kw OR (vomit):ti,ab,kw |
| #13 | (emesis):ti,ab,kw OR (PONV):ti,ab,kw OR (PON):ti,ab,kw OR (POV):ti,ab,kw OR (postoperative nausea):ti,ab,kw |
| #14 | postoperative vomiting |
| #15 | #1 OR #2 OR #3 OR #4 OR #5 OR #6 |
| #16 | #7 OR #8 OR #9 |
| #17 | #10 OR #11 |
| #18 | #12 OR #13 OR #14 |
| #19 | #15 OR #17 |
| #20 | #16 OR #18 |
| #21 | #19 AND #20 |
|  |  |
| Web of Science | |
| # 1 | TOPIC: (acupuncture) OR TOPIC: (moxibustion) OR TOPIC: (electroacupuncture) OR TOPIC: (acupuncture therapy) OR TOPIC: (acupressure) OR TOPIC: (transcutaneous electric nerve stimulation) OR TOPIC: (transcutaneous electric) OR TOPIC: (TEN) OR TOPIC: (TEA)  Databases= WOS, DIIDW, KJD, MEDLINE, RSCI, SCIELO Timespan=All years  Search language=Auto |
| # 2 | TOPIC: (nausea) OR TOPIC: (vomiting) OR TOPIC: (postoperative nausea and vomiting) OR TOPIC: (retching) OR TOPIC: (vomit) OR TOPIC: (emesis) OR TOPIC: (PONV) OR TOPIC: (PON) OR TOPIC: (POV) OR TOPIC: (postoperative nausea) OR TOPIC: (postoperative vomiting)  Databases= WOS, DIIDW, KJD, MEDLINE, RSCI, SCIELO Timespan=All years  Search language=Auto |
| #3 | #1 AND #2 |
|  |  |
| Ovid/Embase | |
| #1 | (acupuncture or moxibustion or electroacupuncture or acupuncture therapy or acupressure or transcutaneous electric nerve stimulation or transcutaneous electric or TEN or TEA).ab. |
| #2 | (nausea or vomiting or (postoperative nausea and vomiting) or retching or vomit or emesis or PONV or PON or POV or postoperative nausea or postoperative vomiting).ab. |
| #3 | #1 AND #2 |
